# Conservative Management of Transitional Cell Carcinoma of the Kidney and Ureter

**DOI:** 10.1155/DTE.1.121

**Published:** 1995

**Authors:** Gregory T. Bales, Edward S. Lyon, Glenn S. Gerber

**Affiliations:** 1 Division of Urology Department of Surgery University of Chicago Hospitals and Clinics USA; 2 Section of Urology University of Chicago Hospitals 5841 S. Maryland Avenue Chicago IL 60637 USA

## Abstract

We report six patients with upper tract transitional cell neoplasms who were treated by an endourologic approach. Two patients had undergone nephroureterectomy previously, whereas one patient had a single functioning kidney. Two patients were deemed candidates for endourologic procedures secondary to significant comorbid disease precluding major open surgery. The final patient had a low-grade distal ureteral lesion and desired a conservative approach.

Of six patients, two were rendered tumor free without recurrence 48 months and 60 months after surgery. Two patients had multiple superficial recurrences managed endoscopically and are currently disease free after 12 and 48 months, respectively. One patient died from metastatic transitional cell cancer 18 months postoperatively whereas one died perioperatively secondary to bleeding and multiple organ failure.

Of five patients who tolerated endoscopic therapy, four were disease free with a mean follow-up of 30 months. Endoscopic treatment of upper urinary tract transitional cell carcinoma is feasible, although the risk of disease recurrence and progression may be significant.


*Diagnostic and Therapeutic Endoscopy*, 1995, Vol. 1, pp. 121–123
Reprints available directly from the publisher
Photocopying permitted by license only

© 1995 Harwood Academic Publishers GmbH
Printed in Malaysia

### Conservative Management of Transitional Cell Carcinoma of the Kidney and Ureter

GREGORY T. BALES, EDWARD S. LYON, and GLENN S. GERBER

*Division of Urology, Department of Surgery, University of Chicago Hospitals and Clinics*

*(Received November 1, 1993; final form January 22, 1994)*

We report six patients with upper tract transitional cell neoplasms who were treated by an endourologic approach. Two patients had undergone nephroureterectomy previously, whereas one patient had a single functioning kidney. Two patients were deemed candidates for endourologic procedures secondary to significant comorbid disease precluding major open surgery. The final patient had a low-grade distal ureteral lesion and desired a conservative approach.

Of six patients, two were rendered tumor free without recurrence 48 months and 60 months after surgery. Two patients had multiple superficial recurrences managed endoscopically and are currently disease free after 12 and 48 months, respectively. One patient died from metastatic transitional cell cancer 18 months postoperatively whereas one died perioperatively secondary to bleeding and multiple organ failure.

Of five patients who tolerated endoscopic therapy, four were disease free with a mean follow-up of 30 months. Endoscopic treatment of upper urinary tract transitional cell carcinoma is feasible, although the risk of disease recurrence and progression may be significant.

**KEY WORDS:** transitional cell cancer, endoscopy, uretoroscopy, ureteral cancer

### INTRODUCTION

The standard treatment of upper tract urothelial tumors is nephroureterectomy with removal of a cuff of bladder surrounding the ipsilateral ureteral orifice (1–3). However, a variety of endourological approaches to renal pelvic and ureteral transitional cell carcinomas have been reported. Some investigators have suggested that conservative endourologic management in selected patients with upper tract urothelial tumors and a normal contralateral kidney may be appropriate (4). In most cases, an endoscopic approach is selected because of tumor in a solitary renal unit, bilateral synchronous tumors, renal insufficiency, or significant comorbid disease that precludes a major open surgical procedure. We have treated six patients recently with renal pelvic and/or ureteral malignancy by a variety of endoscopic procedures, and we herein report our results.

Address for correspondence: Glenn S. Gerber, M.D., Section of Urology, University of Chicago Hospitals, 5841 S. Maryland Avenue, Chicago, IL 60637

### MATERIALS AND METHODS

Six patients with upper tract transitional cell cancer underwent endourologic treatment at the University of Chicago Hospital using a variety of procedures. Patient characteristics are presented in Table 1.

Treatment was selected based on tumor grade and stage, radiographic appearance, status of the contralateral renal unit, and/or presence of significant comorbid disease. Postoperative surveillance was performed every 3 to 12 months using radiographic and/or endoscopic evaluation.

### RESULTS

All patients completed the endourologic procedures successfully. The type of treatment performed and the long-term results are presented in Table 2.

Patient 1 had significant comorbid disease consisting of diabetes mellitus, severe coronary artery disease, and cirrhosis. It was felt that he could not tolerate a major open surgical procedure. After an uneventful percutaneous resection of a renal pelvic tumor, the 30F working sheath

**Table 1** Patient characteristics

| *Patient* | *Age/Sex* | *Presenting Symptoms* | *Intravenous Pyelogram Findings* | *Grade* | *Status of Contralatral Kidney* |
|---|---|---|---|---|---|
| 1 | 73/M | Hematuria | Filling defect, right renal pelvis | II | Nonfunctional |
| 2 | 84/M | Hematuria | Filling defect, right renal pelvis | II | Absent, status post nephroeureterectomy |
| 3 | 82/F | Hematuria | Left UVJ mass with hydronephrosis | II | Normal |
| 4 | 84/M | Follow-up pyelogram | Filling defect, right distal ureter | II | Normal |
| 5 | 57/F | Hematuria | Right renal pelvic mass | II–III | Absent, status post nephroureterectomy |
| 6 | 76/F | Hematuria | Filling defect, right distal ureter | I | Normal |

was replaced by a 24F nephrostomy tube. Severe bleeding from the nephrostomy tract was encountered. This was controlled by reinflation of the fascial balloon dilation catheter within the nephrostomy tract. The patient developed multiorgan failure and died on postoperative day 2.

Patient 2 had a recurrence at 5 months with a higher grade lesion (grade IV). This was treated percutaneously, as was another recurrence 6 months later. The patient died 18 months after his first procedure from widespread malignancy.

Patients 3 and 5 had recurrence of superficial transitional cell cancer in the ureter and renal pelvis, respectively, managed by repeat resection at 6-month follow-up. Both patients have had no further recurrence. Patients 4 and 6 had no further disease after the initial treatment.

There were no complications related to any surgical procedure other than the death of patient 1.

### DISCUSSION

Upper tract transitional cell cancer traditionally has been managed by nephroureterectomy with removal of a cuff of bladder. Parenchymal-sparing conservative surgery has been advocated for selected cases as early as 1948 (5). The endourologic approach to upper tract urothelial tumors may be performed using ureteroscopy or via a percutaneous tract. While several investigators have reported successes with both these modalities, it is generally preferable to use ureteroscopy initially and reserve a percutaneous approach for those tumors that cannot be adequately treated ureteroscopically. This is largely owing to the risk of nephrostomy tract seeding by tumor cells (6). One potential drawback of an endourologic approach may be the difficulty in adequately staging renal pelvic and ureteral tumors leading to understaging and inadequate treatment in some cases. In addition, the risk of local recurrence and disease progression following endourologic treatment may be significant (7).

Endourologic management of upper tract tumors in our series was effective. Five of the six patients completed the procedures successfully. Three of these five underwent endoscopic treatment of recurrent transitional cell tumors noted on routine surveillance studies. Three patients are currently disease free with a fourth having died of unrelated causes with no evidence of disease. Average follow-up of these four patients was 42 months.

There was one perioperative death in a patient with a nonfunctioning contralateral kidney. This patient had severe

**Table 2** Treatment results

| *Patient* | *Treatment* | *Local Recurrence* | *Disease Progression* | *Length of Follow-up* | *Follow-up Status* |
|---|---|---|---|---|---|
| 1 | Percutaneous resection | Not available | Not available | Not available | Not available |
| 2 | Percutaneous resection | 5 months<br>11 months | Yes | 18 months (died) | Metastatic disease |
| 3 | Ureteroscopic resection | 6 months | No | 12 months | Disease free |
| 4 | Ureteroscopic resection | No | No | 60 months (died) | Disease free |
| 5 | Percutaneous resection | 6 months | No | 48 months | Disease free |
| 6 | Ureteroscopic resection | No | No | 48 months | Disease free |

concomitant medical illnesses including diabetes, cirrhosis, and coronary artery disease precluding a major open surgical procedure. Significant nephrostomy tract bleeding was encountered at the end of the procedure, and this ultimately led to multiorgan failure and death. The second death occurred in an 84-year-old patient with a solitary kidney who died 18 months after the initial percutaneous resection of a renal pelvic tumor. In general, tumors of the renal pelvis are larger and more frequently multifocal than those in the ureter. Therefore, disease progression and local recurrence may be seen more often after endourologic treatment of renal pelvic malignancies. This may be an important consideration in determining treatment options for patients with upper tract urothelial tumors.

The endourologic approach to transitional cell cancer of the upper urinary tract in patients with a normal contralateral kidney remains controversial (8,9). While some authors have advocated this approach in those with low-stage, low-grade disease, others feel that this is generally inappropriate. Reasons for this include the risk of local recurrence and disease progression and the need for frequent endoscopic surveillance postoperatively. In general, the vast majority of patients with transitional cell cancer of the upper urinary tract and a normal contralateral kidney should be treated by nephroureterectomy.

In summary, our series supports the feasibility of an endourologic approach to upper urinary tract transitional cell cancer. In selected situations such as those involving renal function, tumor in a solitary kidney, bilateral disease, or significant comorbid illness, endourologic surgery may be preferable. However, our series also demonstrates the significant risk of disease recurrence and progression, which makes frequent postoperative surveillance mandatory. Finally, our single perioperative death is a reminder that conservative, endourologic procedures still may lead to significant complications, particularly in elderly patients with other major medical problems.

### REFERENCES

1. Cummings KB, Nephroureterectomy: rationale in the management of transitional cell carcinoma of the upper urinary tract. *Urol Clin Am*, 7:569, 1980
2. Murphy DM, Zincke H, Furlow WL, Management of high grade transitional cell cancer of the upper urinary tract. *J Urol*, 125:25, 1981.
3. Droller MJ, Transitional cell cancer: upper tracts and bladder. In: Walsh, P. C. Gittes, R. F. Perlmutter A. D. Stamey, T. A. Philadelphia: Campbell's Urology, 5th eds., W. B. Saunders Co., pp. 1343–1440, 1986.
4. Orihuela E, Smith AD, Percutaneous treatment of transitional cell carcinoma of the upper urinary tract. *Urol Clin N A*, 15:425, 1988.
5. Ferris DO, Daut RV, Epithelioma of the pelvis of a solitary kidney treated by electrocoagulation. *J Urol*, 59:577, 1948.
6. Tomera KM, Leary FJ, Zincke J, Pyeloscopy in urothelial tumors. *J Urol*. 127:1088, 1982.
7. Gerber GS, Lyon ES, Endourologic management of upper tract urothelial tumors. *J Urol*, 150:2, 1993.
8. Streem SB, Pontes EJ, Percutaneous management of upper tract transitional cell carcinoma. *J Urol*, 135:773, 1986.
9. Woodhouse CRJ, Kellett MJ, Bloom HJG, Percutaneous renal surgery and local radiotherapy in the management of renal pelvic transitional cell carcinoma. *B J Urol*, 58:245, 1986.

